# Age-at-migration, ethnicity and psychosis risk: Findings from the EU-GEI case-control study

**DOI:** 10.1371/journal.pmen.0000134

**Published:** 2024-10-02

**Authors:** Humma Andleeb, Bettina Moltrecht, Charlotte Gayer-Anderson, Celso Arango, Manuel Arrojo, Giuseppe D’Andrea, Miquel Bernardo, Christina Marta Del-Ben, Lieuwe de Haan, Laura Ferraro, Daniele La Barbera, Erika La Cascia, Pierre-Michel Llorca, Paolo Rossi Menezes, Diego Quattrone, Julio Sanjuán, Jean-Paul Selten, Andrei Szöke, Ilaria Tarricone, Els van der Ven, Eva Velthorst, Bart P. F. Rutten, Peter B. Jones, Craig Morgan, Hannah E. Jongsma, Julian Edbrooke-Childs, James B. Kirkbride

**Affiliations:** 1 PsyLife Group, Division of Psychiatry, UCL, London, United Kingdom; 2 Clinical, Educational and Health Psychology, UCL, London, United Kingdom; 3 Evidence-Based Practice Unit, Anna Freud Centre and UCL, London, United Kingdom; 4 Centre for Longitudinal Studies, Social Research Institute, Institute for Education, UCL, London, United Kingdom; 5 Department of Health Service and Population Research, Institute of Psychiatry, Psychology and Neuroscience, King’s College London, London, United Kingdom; 6 Department of Child and Adolescent Psychiatry, Institute of Psychiatry and Mental Health, Hospital General Universitario Gregorio Maranon, Madrid, Spain; 7 Centre for Biomedical Research in the Mental Health Network, CIBERSAM, Madrid, Spain; 8 School of Medicine, Universidad Complutense de Madrid, Madrid, Spain; 9 University of Montreal Hospital Research Centre (CRCHUM), Montréal, Québec, Canada; 10 Douglas Mental Health University Institute, Prevention and Early Intervention Program for Psychosis (PEPP-Montréal), Montréal, Québec, Canada; 11 Barcelona Clinic Schizophrenia Unit, Hospital Clinic, Departament de Medicina, Institut de Neurociències (UBNeuro), Institut d’Investigacions Biomèdiques August Pi I Sunyer (IDIBAPS), CIBERSAM, ISCIII, Universitat de Barcelona (UB), Barcelona, Spain; 12 Neuroscience and Behavior Departmente, Ribeirão Preto Medical School, University of São Paulo, São Paulo, Brazil; 13 Department of Psychiatry, Early Psychosis Section, Academic Medical Centre, University of Amsterdam, Amsterdam, the Netherlands; 14 Department of Biomedicine, Neurosciences and Advanced Diagnostics, University of Palermo, Palermo, Italy; 15 Centre Hospitalier et Universitaire, Département de Psychiatrie, CNRS, Clermont Auvergne INP, Institut Pascal (UMR 6602), Université Clermont Auvergne, Clermont-Ferrand, France; 16 Department of Preventive Medicine, Faculdade de Medicina, University of São Paulo, São Paulo, Brazil; 17 Social, Genetic and Developmental Psychiatry Centre, Institute of Psychiatry, Psychology and Neuroscience, King’s College London, London, United Kingdom; 18 Hospital Clínico Universitario de Valencia, Universidad de Valencia, Valencia, Spain; 19 Biomedical Research Networking Centre in Mental Health (CIBERSAM), Madrid, Spain; 20 Biomedical Research Institute INCLIVA, Valencia, Spain; 21 School for Mental Health and Neuroscience, University of Maastricht, Maastricht, The Netherlands; 22 Univ Paris Est Creteil, INSERM, IMRB, AP-HP, Hôpitaux Universitaires “H. Mondor”, DMU IMPACT, Fondation Fondamental, Creteil, France; 23 Bologna Transcultural Psychosomatic Team (BoTPT), Department of Medical and Surgical Sciences, University of Bologna, Bologna, Italy; 24 Department of Clinical, Developmental and Neuropsychology, Vrije Universiteit Amsterdam, Amsterdam, the Netherlands; 25 Department of Research, Community Mental Health Service GGZ Noord-Holland-Noord, Heerhugowaard, the Netherlands; 26 Department of Psychiatry and Neuropsychology, School for Mental Health and Neuroscience, Maastricht University Medical Centre, Maastricht, the Netherlands; 27 Department of Psychiatry, Cambridgeshire & Peterborough NHS Foundation Trust, University of Cambridge and CAMEO, Cambridge, United Kingdom; 28 ESRC Centre for Society and Mental Health, Institute of Psychiatry, Psychology, and Neuroscience, King’s College London, London, United Kingdom; 29 Centre for Transcultural Psychiatry “Veldzicht” Balkbrug, Balkbrug, the Netherlands; 30 VR Mental Health Group, University Center for Psychiatry, Univerisity Medical Centre Groningen, Groningen, the Netherlands; Universidad del Valle, COLOMBIA

## Abstract

Several studies have highlighted increased psychosis risk in migrant and minority ethnic populations. Migration before age 18 appears to increase risk, but further evidence is required. We investigated this issue in a European case-control study. We hypothesized that migration during two key socio-developmental periods, childhood and adolescence, would be most strongly associated with increased odds of psychosis, and that this would be more pronounced for racialised minorities. We used data from five countries in the EUropean network of national schizophrenia networks studying Gene-Environment Interactions [EU-GEI] study. We examined the association between migration in infancy (0–4 years), childhood (5–10 years), adolescence (11–17 years) or adulthood (18+ years) and first episode psychotic disorder. We fitted unadjusted and adjusted logistic regression models to estimate odds ratios [OR] and 95% confidence intervals [95%CI] for associations between age-at-migration and psychosis. In stratified models, we also examined whether these associations varied by ethnicity. The sample consisted of 937 cases and 1,195 controls. Migration at all ages, including infancy (OR: 2.03, 95%CI: 1.01–4.10), childhood (OR: 2.07, 95%CI: 1.04–4.14), adolescence (OR: 3.26, 95%CI: 1.89–5.63) and adulthood (OR: 1.71, 95%CI: 1.21–2.41), was associated with increased odds of psychosis compared with the white majority non-migrant group, after adjustment for all confounders except ethnoracial identity. After additional adjustment for ethnoracial identity, only migration during adolescence remained associated with psychosis (OR 1.94, 95%CI: 1.11–3.36). In stratified analyses, migration during adolescence was associated with increased odds of psychosis in Black (OR: 6.52, 95%CI: 3.00–14.20) and North African (OR: 16.43, 95%CI: 1.88–143.51) groups.Migration during adolescence increased psychosis risk, particularly in racially minoritised young people. This suggests that development of interventions for minoritised young migrants that alleviate stressors associated with migration and acculturation are warranted.

## Introduction

Migration is an established risk factor for psychotic disorders [1], and some groups, including first-generation migrants [2] and some racialised minorities [3, 4] experience higher rates of psychosis, with varying but more pronounced rates among those from non-European countries based on country of origin [5]. Elevated rates of psychosis extend to children of migrants, and vary from 1.5 to up to 5 times greater for racialised minorities, depending on ethnicity and context compared with the White majority population in different countries [1].

Growing evidence suggests that exposure to detrimental social determinants of health, including socioeconomic disadvantage, psychosocial disempowerment and childhood trauma may explain a substantial part of this variation [6, 7]. For example, both pre-migration and post-migration social disadvantage have been associated with increased psychosis risk amongst migrant groups [8, 9]. Migrants and their children are more likely to have been exposed to childhood trauma than non-migrants, with one recent study finding that more than a third of first-generation migrants, irrespective of having been diagnosed with first-episode psychosis (FEP), had experienced some form of childhood maltreatment [10]. Further evidence for a possible role of pre-migration trauma as a contributor to psychosis risk in some migrants includes elevated psychosis rates in refugee migrants compared with other migrants from similar regions of origin [11]. Refugees will have been exposed to a variety of potential stressors including war, famine, persecution or other threats to personal safety and security, as well as migration journeys involving displacement, resettlement and asylum seeking. In addition, many migrants and their children are likely to continue to be exposed to a range of social and economic barriers to health that are structurally determined, including discrimination, socioeconomic disadvantage, precarity, and isolation [12]. A recent review highlighted that structural racism at both the neighbourhood and individual level elicits multitude of social disadvantage that shape psychosis risk [13].

Exposure to these social determinants of health may be particularly harmful during childhood and adolescence, which present critical periods for social, cultural and physical development [14, 15]. During this period, and particularly in adolescence, the human brain typically develops by rapidly adapting to new environmental and social experiences [16]. It is possible that traumatic events, including those to which migrants and their children are more likely to be exposed, may disrupt typical neurodevelopment, increasing psychosis risk. People who go on to develop psychosis often exhibit abnormal social and cognitive development, followed by social withdrawal in the prodromal phase (typically emerging in adolescence) [17]. Exposure to pre-migratory trauma during critical windows of neurodevelopment in childhood and adolescence may be disruptive to later mental health. Migration itself may also disrupt social and cognitive development, for example, by interrupting social networks, by affecting identity formation, and/or by additional demands to develop new languages and effective acculturation strategies. For these reasons, migration during childhood and adolescence may present key risk periods for later psychosis risk.

There is some evidence to suggest that migration during childhood and adolescence is associated with increased psychosis risk. For example, a systematic review reported that people who migrated before 18 years old were up to twice as likely to be diagnosed with psychotic disorder than non-migrant populations in the host country [18]. Nonetheless, pinpointing any potential developmental windows during childhood and adolescence when migration may have specific effects on later psychosis risk has proved challenging. Most studies, even when well-designed and large overall, often have small migrant sample sizes resulting in risk estimates by age-at-migration that overlap. Indeed, the authors of the review above concluded there is currently insufficient evidence to determine whether psychosis risk differed by age-at-migration. Previous individual studies have found peak estimates of increased psychosis risk in migrants, usually relative to a White, non-migrant baseline group, to occur in infancy (0–4 years old) [19], middle childhood (5–12 years old) [20] or adolescence (13–18 years old) [21].

Further studies are therefore required to shed light on this issue. We investigated whether migration at different age periods over the life course increased risk of psychotic disorder compared with non-migrant populations, and whether this had the same effect across all ethnic groups. We hypothesised that migration in childhood and adolescence would increase psychosis risk to a greater extent than migration in adulthood and when compared with non-migrants. We also hypothesised that these effects would be more pronounced in migrants from minoritised ethnic groups, given the above evidence of increased rates of psychosis in several minoritised ethnic groups.

## Methods

### Study design

The EU-GEI study is a multicentre, international research collaboration that included an incidence and case-control programme of research on FEP in 17 sites across six countries, in Brazil, England, France, Italy, the Netherlands and Spain [22]. The study followed a standardised protocol across all settings, with regular staff training provided by the central EU-GEI management team via a dedicated training work package [22]. The study recruited participants between 1^st^ May 2010 and 30^th^ April 2015. All incident cases with a FEP were identified in these sites, provided they met standardised inclusion criteria: aged 18–64, living in the catchment areas, and presenting to mental healthcare services with a confirmed International Classification of Diseases, 10^th^ revision [ICD-10], first episode of non-affective (F20-29) or affective psychotic disorder (F30-33), confirmed via a standardised research diagnosis [22]. Those with prior contact with services for psychotic symptoms outside of the study period, onset of psychosis by organic cause or intoxication, severe learning disabilities and insufficient site language fluency were excluded, following detailed case note review and liaison with clinical teams in each setting [22]. For the present paper, we also excluded participants (N = 494) in our Brazilian site, Ribeirão Preto, given little immigration in that setting (N = 2) and the different context in which migration to Brazil occurs compared with our Western Europe sites. Data for this study were drawn from the case-control arm of the study, as described below.

### Ethics statement

Ethical approval was obtained from the local research ethics committees of each participating study site [23]. All procedures relating to this work are in accordance with the ethical standards of the relevant national and institutional committees on human experimentation and with the Helsinki Declaration of 1975, as revised in 2008. All participants gave written informed consent.

### Case recruitment

All incidence cases were invited to participate in a more detailed case-control arm of the study. Cases who gave written informed consent completed a series of assessments to obtain data on genetic, social and clinical factors.

### Control recruitment

Population-based controls were recruited using a combination of random and quota sampling strategies in each site to correspond to the demographic characteristics of the local population by age, self-ascribed ethnoracial identity and sex. Those with prior or current psychotic symptoms were excluded as controls, in addition to the aforementioned inclusion and exclusion criteria. In some sites, some groups were oversampled to enable subgroup analyses [22].

### Exposure

Our exposure variable was age-at-migration, recorded using the Medical Research Council Socio-demographic Schedule (MRC SDS) [24]. We categorised age-at-migration to broad developmental periods coinciding with typical transitions through school in Europe (S1 Table): infancy (0 to 4 years), childhood (5 to 10 years), adolescence (11 to 17 years), and adulthood (18 years and older). We defined non-migrant participants of White ethnoracial identity as the reference group, while non-migrants from any other minoritised ethnic group were coded into separate age-at-migration categories.

### Confounders

**Fig 1:**
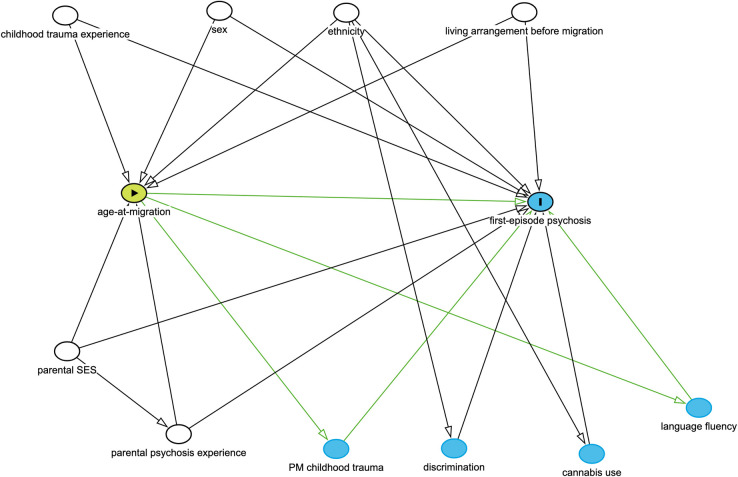
Exposure, outcome, unobserved and observed confounders and mediators summarised in direct acyclic graph (DAG). The proposed causal association between age-at-migration (green oval) and first episode psychosis (blue oval, with “I”). A range of confounders, both adjusted (white ovals) and unadjusted (red ovals), may provide alternative explanations for this association. We blocked the effect of adjusted confounders via regression adjustment in our models. We decided not to control for unadjusted confounders of participant socioeconomic status (SES), discrimination and history of psychosis in family members other than the parents. Participant SES may have been on the causal pathway between age-at-migration and psychosis. Nevertheless, we adjusted for parental SES and parental history of psychosis which would have captured some of the underlying confounding effects that these unobserved constructs represent. Green arrows represent potential causal pathways. We were unable to separate pre- and post-migratory (PM) childhood traumas using our trauma measure (the childhood trauma questionnaire), meaning we may have inappropriately controlled for some post-migratory traumas that were mediators (blue ovals) of the relationship between age-at-migration and psychosis. We did not control for language fluency, which was a potential mediator.

We identified confounding variables informed by the previous literature, and with the input of a lived experience advisory group (S1 Text). We codified this knowledge, and our assumptions about the associations between confounders, exposure and outcome in a directed acyclic graph (DAG) (Fig 1) to identify a necessary set of confounders to include in our analyses. These included ethnoracial identity, which we coded into six categories based on self-ascribed ethnic identity: White, Black, Mixed, Asian, North African, and Other, and sex (male/female). We also controlled for parental social class and living arrangements prior to migration, as we have previously shown these markers of social disadvantage are strongly associated with psychosis risk amongst migrants [9]. Parental social class (highest) was based on parental occupation, coded into professional, intermediate and working class occupations or long-term unemployed. Living arrangements prior to migration were coded as living alone, with family, or other. For migrants, living arrangements prior to migration were used, and for non-migrants living arrangements five years prior to assessment were used. In addition, we controlled for parental history of either psychosis or any other mental illness were coded as binary variables (yes/no), measured using the Family Interview for Genetic Studies (FIGS) [25], and childhood trauma, estimated using the total score on the Childhood Trauma Questionnaire (CTQ) [26], and grouped into quartiles.

### Missing data

There was no missing outcome data. We quantified levels of missingness on the exposure and confounders and compared the complete-case sample to those with missing data on one or more variables. Missing data were assumed to be at least missing at random (MAR). We imputed missing data using Multiple Imputation by Chained Equations (MICE), a widely used approach to address missing data [27, 28], by estimating imputed values of the missing data based on appropriate regression models using observed covariates and auxiliary variables [29]. In addition to all aforementioned covariates and outcome status, we included region of birth, EU-GEI site, self-rated fluency in the majority language in each setting, and age-at-first-contact as auxiliary variables in our imputation models (see S2 Text). Age-at-migration terms were created following multiple imputation using passive imputation methods (see S2 Text for full details). Fifty datasets were imputed and analyses were conducted using these datasets according to Rubin’s Rules [30].

### Statistical analyses

We provided descriptive statistics of sample and missing data characteristics, using Pearson’s correlation X^2^ tests and Fisher’s exact tests, as appropriate. We then fitted four multilevel logistic regression models (random intercepts models with participants nested within sites) to the imputed data, as follows:

- Unadjusted model: Odds ratios were calculated for the crude association between age-at-migration and case-control status
- Adjusted Model 1: Adjusted for sex, parental social class, living arrangements, history of parental psychosis, history of parental experience of other mental illness and childhood trauma
- Fully-adjusted Model 2: Adjusted Model 1 plus ethnoracial identity. Since ethnoracial identity may also have influenced age-at-migration, and is known to be associated with variation in psychosis risk [20], we adjusted for this variable in this model to examine its potential effect on this association

To investigate whether the association between age-at-migration and psychosis differed by ethnoracial identity, we also ran stratified analyses in Model 2 to estimate the effect of age-at-migration for each ethnoracial minority group relative to the White majority non-migrant reference. No formal test of interaction between age-at-migration and ethnoracial identity was possible due to the reference group for both variables being perfectly collinear (White majority non-migrant group for age-at-migration and the white group for the ethnoracial identity variable). All models were fitted with robust standard errors using the Huber/White/sandwich estimator, allowing us to relax assumptions about normality and identical distribution of errors. We compared the results following multiple imputation with those obtained from complete case analyses. We reported odds ratios [OR] and their 95% confidence intervals [95% CI]. All statistical analyses were conducted in Stata 17 [31].

## Results

### Sample

Case-control data was available for 2,133 participants, of which one case experienced FEP before migration and was excluded. The final sample (n = 2,132) consisted of 937 FEP cases (44.0%) and 1195 (56.0%) controls.

### Missing data

Overall, 28.5% of the sample had at least one missing exposure or covariate, although only 9.6% of the sample were missing data on three or more variables (S2 Table, S1 Fig). Missingness ranged from no participants missing data on ethnoracial identity and sex to 15.2% of participants on parental history of psychosis. Participants with missing data were more likely to be cases (p<0.001), aged 25–44 years old (p = 0.02), migrate before 18 years old (p<0.001), be born outside of Europe (p<0.001), not be fluent in the main language of the country of residence (p = 0.007), be from working class parents (p<0.001) and have a parental history of psychosis (p = 0.03) than complete cases, but did not differ by sex (p = 0.36), ethnoracial identity (p = 0.33), living arrangements (p = 0.45), parental history of any other mental illness (p = 0.34) or childhood trauma (p = 0.76) (S2 Table).

### Sample characteristics

In the complete case sample (N = 1,525), a higher proportion of cases compared with controls migrated at each age-of-migration (Table 1), were men (62.7% vs. 47.0%), were aged less than 45 years old (87.3% vs. 69.5%), were from an ethnic minority background (34.0% vs. 17.6%), had working class parents (40.7% vs. 33.6%), lived with others rather than family or alone (28.1% vs 22.5%), had a parental history of psychosis (7.6% vs 1.8%) or other mental illness (15.9% vs. 12.6.6%), and belonged to the highest quartile of childhood trauma (39.0% vs. 16.1%) (all p<0.01, Table 1). The distribution of cases and controls differed by age-at-migration category (p<0.001), with cases less likely to be in the White majority non-migrant groups (59.5% vs. 74.7%) than controls, and more likely to be any age-at-migration group (Table 1).

**Table 1:** Sample characteristics by case-control status.

	*Subject status N (%)*	
	*Cases*	*Controls*
Total^1^	937 (43.9)	1,195 (56.1)
***Age-of-migration***	***Χ***^***2***^ ***= 66*.*5 (5)***	***p<0*.*001***
White majority non-migrant	548 (59.5)	887 (74.7)
Infancy (0–4 years)	26 (2.8)	23 (1.9)
Childhood (5–10 years)	38 (4.1)	25 (2.1)
Adolescence (11–17 years)	46 (5.0)	23 (1.9)
Adulthood (18–64 years)	129 (14.0)	139 (11.7)
Ethnic-minority non-migrant	134 (14.6)	91 (7.7)
*Missing* ^*1*^	*16 (1*.*7)*	*7 (0*.*6)*
***Sex***	***Χ***^***2***^ ***= 52*.*3 (1)***	***p<0*.*001***
Male	588 (62.7)	562 (47.0)
Female	349 (37.3)	633 (53.0)
*Missing* ^*1*^	*0 (0*.*0)*	*0 (0*.*0)*
***Age group*** ^*2*^	***Χ***^***2***^ ***= 114*.*3 (4)***	***p<0*.*001***
18–24 years	333 (35.4)	268 (22.5)
25–34 years	312 (33.3)	350 (29.3)
35–44 years	173 (18.5)	212 (17.8)
45–54 years	89 (9.5)	219 (18.3)
55–64 years	30 (3.2)	145 (12.1)
*Missing* ^*1*^	*0 (0*.*0)*	*1 (0*.*1)*
***Place of birth*** ^*2*^	***Χ***^***2***^ ***= 49*.*4 (10)***	***p<0*.*001***
France	73 (7.8)	124 (10.4)
Italy	158 (16.9)	258 (21.6)
Spain	170 (18.1)	186 (15.6)
The Netherlands	147 (15.7)	184 (15.4)
UK	150 (16.0)	241 (20.2)
Other Europe	28 (3.0)	35 (2.9)
Asia & Australasia	22 (2.4)	23 (1.9)
Sub-Saharan Africa	71 (7.6)	42 (3.5)
North Africa & Middle East	34 (3.6)	16 (1.3)
Americas	62 (6.6)	71 (5.9)
Other	22 (2.3)	15 (1.3)
*Missing* ^*1*^	*0 (0*.*0)*	*0 (0*.*0)*
***Ethnoracial identity***	***Χ***^***2***^ ***= 77*.*7 (5)***	***p<0*.*001***
White	618 (66.0)	982 (82.2)
Black	152 (16.2)	100 (8.4)
Mixed	47 (5.0)	35 (2.9)
Asian	33 (3.5)	30 (2.5)
North African	52 (5.6)	24 (2.0)
Other	35 (3.7)	24 (2.0)
*Missing* ^*1*^	*0 (0*.*0)*	*0 (0*.*0)*
***Fluency (binarized)*** ^*2*^	***Χ***^***2***^ ***= 27*.*3 (1)***	***p<0*.*001***
Yes	743 (83.5)	1,047 (91.1)
No	147 (16.5)	102 (8.9)
*Missing* ^*1*^	*47 (5*.*0)*	*46 (3*.*9)*
***Parental social class***	***Fisher’s Exact*:**	***p<0*.*001***
Professional	240 (29.4)	406 (36.7)
Intermediate	230 (28.2)	325 (29.4)
Working Class	332 (40.7)	372 (33.6)
Long-term unemployed	14 (1.7)	3 (0.3)
*Missing* ^*1*^	*121 (12*.*9)*	*89 (7*.*5)*
***Living arrangements prior to migration/five years before migration***	***Χ***^***2***^ ***= 9*.*7 (2)***	***p = 0*.*008***
Alone	76 (8.9)	124 (11.4)
Family	542 (63.1)	722 (66.2)
Other	241 (28.1)	245 (22.5)
*Missing* ^*1*^	*78 (8*.*3)*	*104 (8*.*7)*
**Parental history of psychosis**	***Χ***^***2***^ ***= 36*.*2 (1)***	***p<0*.*001***
No	713 (92.4)	1,017 (98.2)
Yes	59 (7.6)	19 (1.8)
*Missing* ^*1*^	*165 (17*.*6)*	*159 (13*.*3)*
***Parental experience of other mental illness***	***Χ***^***2***^ ***= 6*.*8 (1)***	***p = 0*.*009***
No	555 (70.4)	792 (75.9)
Yes	233 (29.6)	252 (24.1)
*Missing* ^*1*^	*149 (15*.*9)*	*151 (12*.*6)*
***Childhood trauma (CTQ score range)***	***Χ***^***2***^ ***= 209*.*5 (5)***	***p<0*.*001***
Quartile 1 (25–28)	101 (12.1)	372 (31.5)
Quartile 2 (29–33)	155 (18.6)	345 (29.2)
Quartile 3 (34–42)	252 (30.3)	276 (23.3)
Quartile 4 (43–102)	325 (39.0)	190 (16.1)
*Missing* ^*1*^	*104 (11*.*1)*	*12 (1*.*0)*

Χ^2^: Pearson’s Chi^2^ test

^1^Item-level missingness, not included in Χ^2^-test comparisons. Percentages reported for other levels of this variable expressed as percentage of those with complete data on this variable

^2^Presented for descriptive purposes and included as auxiliary variables during multiple imputation by chained equations, but not included as part of the covariate adjustment set

### Logistic regression analyses

Following multiple imputation, we observed elevated odds of psychosis with all age-at-migration groups compared with the White majority, non-migrant group, both in an unadjusted model and a model adjusted for all covariates except ethnoracial identity (Model 1, Table 2). In these models, the highest point estimate of increased odds of psychosis relative to the White majority, non-migrant group was associated with migration during adolescence (i.e. adjusted OR [aOR]: 3.26, 95%CI: 1.89–5.63), although confidence intervals overlapped with estimates for other age-at-migration groups. Further adjustment for ethnoracial identity (Model 2, Table 2) substantially attenuated the association between age-at-migration and psychosis, but migration in adolescence remained associated with increased odds of psychosis (aOR: 1.94, 95%CI: 1.11–3.36).

**Table 2:** Unadjusted and adjusted odds ratios for the association between age-at-migration and psychosis, following multiple imputation.

	Odds ratios (95% confidence interval)		
	*Unadjusted OR*	*Adjusted Model 1* ^1^	*Fully-adjusted Model 2* ^2^
***Age at migration***			
White majority non-migrant	1	1	1
Infancy (0 to 4 years)	2.34 (1.42–3.89)*	2.03 (1.01–4.10)*	1.35 (0.81–2.24)
Childhood (5 to 10 years)	2.64 (1.46–4.78)*	2.07 (1.04–4.14)*	1.32 (0.53–3.27)
Adolescence (11 to 17 years)	3.72 (2.08–6.64)*	3.26 (1.89–5.63)*	1.94 (1.11–3.36)*
Adulthood (18 to 64 years)	1.61 (1.19–2.17)*	1.71 (1.21–2.41)*	1.17 (0.71–1.95)
Ethnic minority non-migrant	2.70 (2.34–3.11)*	2.25 (1.87–2.72)*	1.14 (0.61–2.13)
***Sex***	*** ***		
Male	-	1	1
Female	-	0.51 (0.43–0.60)*	0.52 (0.44–0.61)*
***Ethnoracial identity***	*** ***		
White	-	-	1
Black	-	-	2.08 (1.21–3.55)*
Mixed	-	-	1.90 (0.55–6.60)
Asian	-	-	1.58 (0.75–3.33)
North African	-	-	3.23 (1.07–9.79)*
Other	-	-	1.44 (0.61–3.40)
***Parental social class***	*** ***		
Professional	-	1	1
Intermediate	-	1.15 (0.90–1.46)	1.13 (0.89–1.42)
Working Class	-	1.43 (1.02–1.99)*	1.40 (1.01–1.95)*
Long-term unemployed	-	5.07 (1.26–20.44)*	5.00 (1.24–20.12)*
***Living arrangements***	*** ***		
Family	-	1	1
Alone	-	0.74 (0.51–1.07)	0.77 (0.52–1.12)
Other	-	1.44 (0.95–2.18)	1.47 (0.97–2.24)
***Parental psychosis experience***	*** ***		
No	-	1	1
Yes	-	4.00 (2.28–7.01)*	4.13 (2.40–7.12)*
***Parental other mental health experience***	*** ***		
No	-	1	1
Yes	-	1.43 (1.02–1.99)*	1.46 (1.06–2.02)*
***Childhood trauma experience***	*** ***		
Quartile 1 (25–28)	-	1	1
Quartile 2 (29–33)	-	1.65 (1.20–2.26)*	1.66 (1.19–2.30)*
Quartile 3 (34–42)	-	3.22 (2.39–4.34)*	3.21 (2.40–4.30)*
Quartile 4 (43–102)	-	6.30 (4.65–8.55)*	6.28 (4.66–8.45)*

Results stratified by major ethnic groups are presented in Table 3. Small sample sizes for several ethnic groups by age-at-migration led to statistical uncertainty around several odds ratios. Nonetheless, we had sufficient power to demonstrate that people of Black ethnic backgrounds faced elevated odds of psychosis relative to the White majority, non-migrant population across all age-at-migration groups, again peaking with migration in adolescence (aOR: 6.52, 95%CI: 3.00–14.20), but also extending to non-migrant Black groups (aOR: 2.16, 95%CI: 1.32–3.53). We also found evidence that migration in adolescence was associated with the largest increased odds of psychosis for migrants of North African origin (aOR: 16.43, 95%CI: 1.88–143.51) albeit with high imprecision given the small sample size (n = 7). We also observed elevated odds of psychosis associated with migration in adulthood for migrants of North African origin (aOR: 3.28, 95%CI: 1.16–9.30), and for non-migrant participants from mixed ethnic backgrounds (aOR: 3.29, 95%CI: 1.32–3.53) relative to the White majority, non-migrant group.

**Table 3:** Adjusted odds ratios for the association between age-at-migration and psychosis, following multiple imputation and stratified by ethnoracial identity.

***Effect sizes by age-at-migration***	**White** **aOR (95% CI)** ^**1**^	**Black** **aOR (95% CI)** ^**1**^	**Mixed** **aOR (95% CI)** ^**1**^	**Asian** **aOR (95% CI)** ^**1**^	**North African** **aOR (95% CI)** ^**1**^	**Other** **aOR (95% CI)** ^**1**^
White majority non-migrant	1	1	1	1	1	1
Infancy (0 to 4 years)	1.20 (0.32–4.43)	**3.19 (1.74–5.84)** *	1.44 (0.19–10.95)	1.14 (0.33–3.93)	-	1.74 (0.40–7.50)
Childhood (5 to 10 years)	1.68 (0.48–5.81)	**1.94 (1.09–3.44)** *	3.12 (0.41–23.73)	2.58 (0.17–27.69)	3.71 (0.32–43.47)	-
Adolescence (11 to 17 years)	2.05 (0.85–4.96)	**6.52 (3.00–14.20)** *	0.88 (0.23–3.44)	1.29 (0.56–2.91)	**16.43 (1.88–143.51)** *	1.87 (0.26–13.27)
Adulthood (18 to 64 years)	1.10 (0.63–1.95)	**2.69 (1.66–4.34)** *	0.93 (0.29–2.92)	3.03 (0.87–10.62)	**3.28 (1.16–9.30)** *	1.60 (0.59–4.34)
Ethnic minority non-migrant	-	**2.16 (1.32–3.53)** *	**3.29 (1.53–7.07)** *	1.80 (0.72–4.49)	3.63 (0.81–16.24)	1.49 (0.35–6.34)
***Cases by age-at-migration*** ^2^	**N (%)**	**N (%)**	**N (%)**	**N (%)**	**N (%)**	**N (%)**
White majority non-migrant	548 (89.1)	**-**	**-**	-	-	-
Infancy (0 to 4 years)	9 (1.5)	13 (8.8)	2 (4.4)	2 (6.1)	3 (6.4)	4 (11.8)
Childhood (5 to 10 years)	9 (1.5)	14 (9.5)	2 (4.4)	1 (3.0)	2 (4.3)	3 (8.8)
Adolescence (11 to 17 years)	7 (1.1)	21 (14.3)	2 (4.4)	5 (15.2)	7 (14.9)	4 (11.8)
Adulthood (18 to 64 years)	42 (6.8)	38 (25.9)	4 (8.9)	13 (39.4)	14 (29.8)	18 (52.9)
Ethnic minority non-migrant	0 (0.0)	61 (41.5)	35 (77.8)	12 (36.4)	21 (44.7)	5 (14.7)

CI: confidence interval; aOR: adjusted odds ratio

*p<0.05, **bold**; ^p = 0.054; ^#^p = 0.073

^1^Adjusted for gender, parental social class, living arrangement before migration (for migrants) and five years prior to assessment (non-migrants), parental psychosis experience and parental other mental health experience

^2^16 of 937 cases missing age-at-migration omitted from descriptive sample

To examine whether psychosis odds associated with migration during adolescence were distinct from risk associated with other age-at-migration periods, we reparameterised our main models (Table 2) and stratified models for the Black and North African groups (Table 3), setting migration during adolescence as the reference group (S3 Table). For the whole sample, we found evidence that the odds of psychosis associated with migration in adolescence were higher than those associated with migration during adulthood (reflected by lower odds in those who migrated during adulthood: aOR: 0.52, 95%CI: 0.28–0.98), after adjustment for all confounders except ethnoracial identity. Trends in this direction were observed for Black (aOR: 0.41, 95%CI: 0.17–1.03; p = 0.057) and North African (aOR: 0.20, 95%CI: 0.04–1.09; p = 0.062) groups separately with respect to lower odds associated with adulthood migration compared with adolescent migration. Psychosis odds were also lower for non-migrant Black (aOR: 0.33, 95%CI: 0.11–0.91) and North African (aOR: 0.22, 95%CI: 0.05–0.91) groups, and for Black groups who migrated in childhood (aOR: 0.30, 95%CI: 0.12–0.74), than their counterparts who migrated during adolescence.

We conducted sensitivity analyses in our complete case sample (S4 Table), which led to similar odds ratios to those reported from our imputed sample, albeit with the expected greater uncertainty around some estimates reflecting the smaller complete case sample (N = 1,525). This suggested that our imputation model provided a valid representation of the missing data mechanisms underpinning our dataset, lending validity to the results observed from these models.

## Discussion

### Main findings

We found that migration at any age was associated with increased odds of psychosis (OR range 1.71 to 3.26). The greatest increase was evident for those who migrated during adolescence. These findings persisted after adjustment for multiple potential confounders, with evidence that increased risk associated with migration during adolescence was not fully confounded by ethnoracial identity. When stratified by ethnoracial identity, our analyses suggested that migration during adolescence was particularly strongly associated increased odds of psychosis for people from Black and North African backgrounds, albeit sometimes with wide confidence intervals.

### Comparison with the previous literature

A previous meta-analysis reported that migrants who moved prior to 18 years old were twice as likely to be diagnosed with a psychotic disorder than non-migrants [18], but reported similar effect sizes (pooled incidence rate ratios ranged from 1.67 to 1.85 with overlapping confidence intervals) for migration in infancy, early childhood, middle childhood and adolescence. Our findings were partially consistent with this observation, insofar as our unadjusted results showed comparable effects to the unadjusted results upon which that meta-analysis’ findings were derived. In our fully-adjusted models, we extended this research to show that ethnoracial identity had a strong attenuating effect on these associations, as similarly observed elsewhere [32], with only an association between migration during adolescence and psychosis remaining in our work. Our results also extend the previous literature by showing that the effect of migration during adolescence on psychosis risk was most pronounced for people from Black and North African backgrounds, two ethnic groups who have historically faced the grossest inequalities in risk of psychotic disorder [4, 5, 33]. For these groups, we found demonstrable evidence that psychosis risk associated with migration during adolescence was higher than for migration earlier in childhood, or for non-migrant participants in our study of Black and North African heritage (S3 Table).

### Meaning of the findings

Long term migration is considered to be a stressful life event, particularly so during vulnerable developmental periods and for minoritised groups. If adolescent migration represents a specific period of vulnerability that increases later psychosis risk, there may be several plausible theories to account for this. First, adolescent migrants may have accumulated exposure to socioeconomic disadvantage and traumatic life events (parental separation, economic instability violence, interpersonal or civil conflict, persecution) prior to migration that increase psychosis risk [10]. Migrants at this age are likely to have been exposed to such pre-migratory environmental risk factors to a greater extent than their peers who migrated at younger ages [15].

Second, those who migrate in adolescence may face more barriers to successful acculturation than those who migrate at younger ages. For example, adolescents may face more language barriers after migration, compared with those who migrate in infancy or childhood, who have greater opportunities to acquire a second language during childhood development [34]. In this sample, we have previously shown that linguistic difficulties experienced by migrants are associated with increased psychosis risk amongst migrants [6]; our results raise the possibility that language barriers for those who migrate during adolescence may contribute to acculturative stress relevant to risk of later mental health problems during adolescence.

Adolescence is also a sensitive period of social and neuro- development, critical for establishing our sense of self [35]. Structural and functional changes in the brain during this period are thought to be influenced by the social environment around us [14]. During this period, adolescents begin to spend more time with their friends and less time with their family [36] as they seek to develop their own identity. In the context of migration, most people who migrate before 18 years old will do so with their parents and/or caregivers, and may have little autonomy in this decision-making process. Their existing social networks may be disrupted and as such they are less likely to be surrounded by peers and friends they had formed relationships with in their country of origin [37]. They may face difficulties in establishing new peer networks on arrival, particularly in the context of additional barriers around language, culture or minoritised ethnic status [38]. These intersectional experiences may further contribute to increases in acculturative stress for adolescent migrants, including social isolation, loneliness or discrimination, and all such factors have been linked to psychosis risk in previous studies [6], albeit predominantly with cross-sectional designs [39].

Visible minorities experience discrimination, racism and worse mental health outcomes, observed in both first- and second-generation migrants [2]. The extent to which one issue, over-diagnosis (vis-à-vis elevated psychosis risk), contributes to raised rates of psychotic disorder amongst some migrant and ethnic minority group remains unclear. Nonetheless, there is growing evidence that the social determinants of psychosis risk, including trauma [11, 40], socioeconomic disadvantage, sociocultural disempowerment [6] and structural racism [6] disproportionately affect these groups and account for much of the excess risk of psychosis. Moreover, it is unclear how over-diagnosis would explain differential psychosis risk for adolescent migrants, as observed here.

While further studies are required to determine the interplay between migration during adolescence, acculturative stress and psychosis, our findings suggest that healthcare professionals should pay particular action to the mental and behavioural health of young people who migrate in adolescence, particularly for some minoritised ethnic groups known to be at higher risk of psychosis. This could warrant selective prevention strategies for such high risk groups, which may include acculturative support, mental health literacy, psychosocial interventions, or even early detection approaches.

### Strengths and limitations

We used a large case-control dataset from the EU-GEI study, which included participants from multiple, diverse sites and countries with well-characterised data on multiple variables including age-at-migration and several relevant confounders. A further strength of this study was that the analysis plan and interpretation of results was informed by the experience of a lived experience advisory group made up of migrants with experience of psychosis and/or other mental health problems. The group informed the identification of confounders in the analysis plan and decision-making around potential confounding variables to include in the models. This allowed the study team to gain specific insight into how psychosis and mental health are experienced by the public and how epidemiological research can benefit from involving lived experience participation.

This paper also had several limitations. Those who experienced FEP and chose not to participate in this study may have differed in important, unobserved ways to those who took part. It is also possible that our results do not generalise to all migrant and ethnic minority groups. Participants may have differed in important ways to eligible cases or controls who chose not to take part in our study, including by severity of illness (for cases), or demographic and social factors (for all participants). Further, even when generalisable to the target populations in our study, we studied a restricted range of settings in Western Europe; our results may not generalise to migrants to other contexts [18]. On this issue, we relied on broad ethnoracial groupings to enable comparisons by major ethnic group across diverse settings with differential patterns of immigration, and our results may not generalise to specific ethnoracial groups. Our results may also not generalise to refugee migrants, who may have less access to mental health services and who will have experienced more trauma before and potentially during and after immigration; refugees are known to be at increased risk of psychosis compared with both non-refugee migrants from similar regions of origin and the majority population in their host country [11].

We used multiple imputation by chained equations to impute missing data, including important auxiliary variables. Our imputed results were comparable with our complete case results, arguing against substantial bias in the missing data mechanisms in our dataset. While 28.5% of our sample had at least some missing data relevant to our analyses, the proportion of missing data should not be used to guide decisions on multiple imputation [41]. Although multiple imputation allowed us to recover power in this study, some sample sizes for specific age-at-migration groups remained small and led to statistical uncertainty in some of our stratified estimates, including wide confidence intervals in the North African sample. The original EU-GEI case-control study was designed to have 80% power to detect odds ratios of at least two for an environmental exposure [22]. Our study was able to detect such odds ratios, albeit in a smaller sample than originally estimated (81% of the original powered sample [22]); our study would have had less power to detect smaller effect sizes by age-at-migration, particularly in analyses stratified by ethnoracial identity. Larger, powered longitudinal studies in populations with strong ethnic and migrant diversity are required to estimate the odds of psychotic disorder by age-at-migration in different ethnic groups. We were unable to run a formal interaction test for effect modification between age-at-migration and ethnoracial identity on psychosis risk due to perfect collinearity in the reference category for both these variables (i.e. the white majority non-migrant group).

We included childhood trauma as an *a priori* confounder, although based on how it was ascertained, we could not separate traumas that occurred before migration (i.e. confounders) from those that may have occurred after (i.e. mediators). We may therefore have inappropriately adjusted for some childhood traumas on the causal pathway, which would have conservatively biased our estimated odds ratios of the association between age-at-migration and psychosis toward the null. We did not include region of birth as a confounder in our analyses as it was highly correlated with ethnoracial identity and EU-GEI setting, nor fluency which could have been on the causal pathway between age-at-migration and psychosis (Fig 1).

## Conclusion

Our work suggests that migration in adolescence is a strong risk factor for psychosis, particularly for in migrants from certain ethnoracial minority groups. Further work is required to identify the potential biopsychosocial factors that may contribute to this risk during a sensitive period of social, neuro- and identity development.

## Supporting information

S1 Fig Histogram of frequency of missing data across exposure, confounder or auxiliary variables^1^. 1608 of 2,132 participants (28.5%) were missing data on at least one missing exposure, confounder or auxilliary variable. Eleven variables (exposure n = 1; confounder n = 7; auxilliary n = 3) were included in this paper (see S1 Table) for variable-level missingness).
(DOCX)

S1 Table School transition dates in participating European settings. ^1^Mandatory education age is from 3–16 years old, but with vocational training or further education required until 18 years old. ^2^Mandatory education age is 6–16 years old, with upper high school commencing approximately at 14 years old. Students who wish to go on to complete higher education must complete 5 years of upper high school education. ^3^Mandatory education age is 5–16 years old, but a pupil must attend some form of education at least two days a week from 16–18 years old.^4^Mandatory education age is 6–16 years old, with two optional years of post-16 education.^5^Mandatory education age is from 5–18 years old, with mandatory schooling from 5–16 years old.
(DOCX)

S2 Table Sample characteristics by complete case status. Χ^2^: Pearson’s Chi^2^ test. ^1^Participants who were missing on at least one exposure or covariate. ^2^Item-level missingness, not included in Χ^2^-test comparisons. Percentage is percentage of all participants (N = 2,132) missing data on that item. ^3^Presented for descriptive purposes and included as auxiliary variables during multiple imputation by chained equations, but not included as part of the covariate adjustment set.
(DOCX)

S3 Table Reparameterization of selected logistic regression models with migration during adolescence set as the reference group. ^1^Adjusted for gender, parental social class, living arrangement before migration (for migrants) and five years prior to assessment (non-migrants), parental psychosis experience and parental other mental health experience. ^2^Model 1 + adjustment for ethnoracial identity. *p<0.05, **bold.** ^#^p = 0.057. ^^^p = 0.062.
(DOCX)

S4 Table Multivariable regression results from complete case analyses (N = 1,525). ^1^Adjusted for gender, parental social class, living arrangement before migration (for migrants) and five years prior to assessment (non-migrants), parental psychosis experience and parental other mental health experience. ^2^Model 1 + adjustment for ethnoracial identity. *p<0.05, **bold**. ^#^p = 0.053. ^^^p = 0.057.
(DOCX)

S1 Text Lived Experience Advisory Group (LEAP) Input in study. (DOCX)

S2 Text Missing data. (DOCX)
