# Supplementary material for: Age-at-migration, ethnicity and psychosis risk: Findings from the EU-GEI case-control study
Source: PLOS Ment Health. 2024 Oct 2;1(5):e0000134. doi: 10.1371/journal.pmen.0000134 (PMC12798472; doi:10.1371/journal.pmen.0000134)
Supplement: S1 Fig — 1608 of 2,132 participants (28.5%) were missing data on at least one missing exposure, confounder or auxilliary variable. Eleven variables (exposure n = 1; confounder n = 7; auxilliary n = 3) were included in this paper (see S1 Table) for variable-level missingness). (DOCX) [file pmen.0000134.s001.docx]

**S1 Fig: Histogram of frequency of missing data across exposure, confounder or auxiliary variables^1^**

**
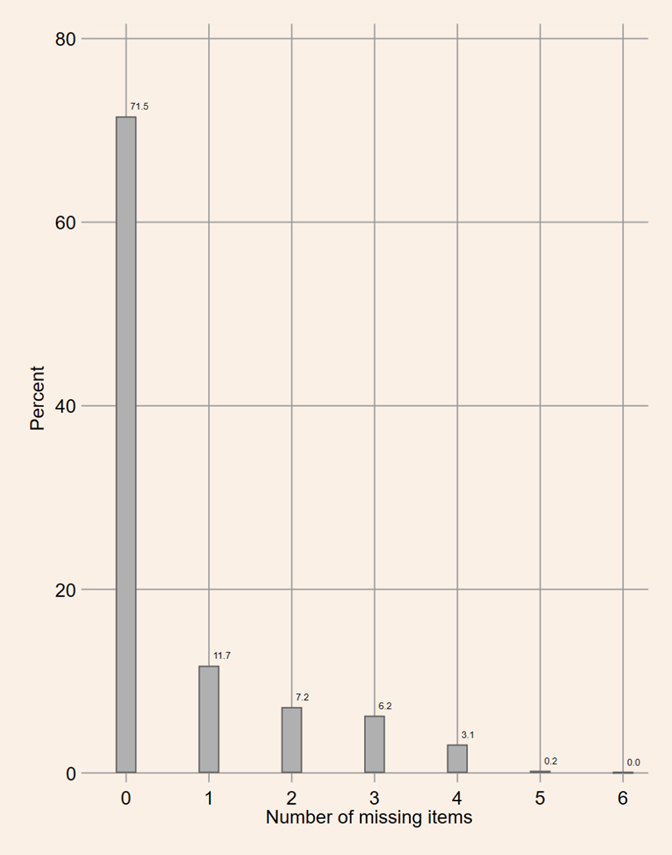
**

^1^608 of 2,132 participants (28.5%) were missing data on at least one missing exposure, confounder or auxilliary variable. Eleven variables (exposure n=1; confounder n=7; auxilliary n=3) were included in this paper (see Table 1, Supplemental Table 1 for variable-level missingness)
