# Supplementary material for: Age-at-migration, ethnicity and psychosis risk: Findings from the EU-GEI case-control study
Source: PLOS Ment Health. 2024 Oct 2;1(5):e0000134. doi: 10.1371/journal.pmen.0000134 (PMC12798472; doi:10.1371/journal.pmen.0000134)
Supplement: S1 Table — 1Mandatory education age is from 3–16 years old, but with vocational training or further education required until 18 years old. 2Mandatory education age is 6–16 years old, with upper high school commencing approximately at 14 years old. Students who wish to go on to complete higher education must complete 5 years of upper high school education. 3Mandatory education age is 5–16 years old, but a pupil must attend some form of education at least two days a week from 16–18 years old.4Mandatory education age is 6–16 years old, with two optional years of post-16 education.5Mandatory education age is from 5–18 years old, with mandatory schooling from 5–16 years old. (DOCX) [file pmen.0000134.s002.docx]

**S1 Table: School transition dates in participating European settings**

| **Country** | **Primary education** | **Secondary education** | **Higher education** |
| --- | --- | --- | --- |
| **France**^1^ | 3-11 years | 11-18 years | 18+ years |
| **Italy**^2^ | 6-11 years | 11-19 years | 19+ years |
| **The Netherlands**^3^ | 5-12 years | 12-18 years | 18+ years |
| **Spain**^4^ | 6-12 years | 12-18 years | 18+ years |
| **UK**^5^ | 5-10 years | 11-18 years | 18+ years |

^1^Mandatory education age is from 3-16 years old, but with vocational training or further education required until 18 years old

^2^Mandatory education age is 6-16 years old, with upper high school commencing approximately at 14 years old. Students who wish to go on to complete higher education must complete 5 years of upper high school education

^3^Mandatory education age is 5-16 years old, but a pupil must attend some form of education at least two days a week from 16-18 years old

^4^Mandatory education age is 6-16 years old, with two optional years of post-16 education

^5^Mandatory education age is from 5-18 years old, with mandatory schooling from 5-16 years old
