# Supplementary material for: Age-at-migration, ethnicity and psychosis risk: Findings from the EU-GEI case-control study
Source: PLOS Ment Health. 2024 Oct 2;1(5):e0000134. doi: 10.1371/journal.pmen.0000134 (PMC12798472; doi:10.1371/journal.pmen.0000134)
Supplement: S2 Table — Χ2: Pearson’s Chi2 test. 1Participants who were missing on at least one exposure or covariate. 2Item-level missingness, not included in Χ2-test comparisons. Percentage is percentage of all participants (N = 2,132) missing data on that item. 3Presented for descriptive purposes and included as auxiliary variables during multiple imputation by chained equations, but not included as part of the covariate adjustment set. (DOCX) [file pmen.0000134.s003.docx]

**S2 Table: Sample characteristics by complete case status**

|  | ***Missing^1^*** | ***Complete cases*** |
| --- | --- | --- |
| Total sample | 607 (28.5) | 1,525 (71.5) |
| ***Case-control status*** | *Χ****^2^=24.7*** | ***p<0.001*** |
| Cases | 349 (37.3) | 588 (62.7) |
| Controls | 258 (21.6) | 937 (78.4) |
| *Missing^2^* | *0 (0.0)* | *0 (0.0)* |
| ***Age-at-migration*** | *Χ****^2^=24.7*** | ***p<0.001*** |
| White majority non-migrant | 377 (26.3) | 1,058 (73.7) |
| Infancy (0-4 years) | 19 (38.8) | 30 (61.2) |
| Childhood (5-10 years) | 26 (41.3) | 37 (58.7) |
| Adolescence (11-17 years) | 31 (44.9) | 38 (55.1) |
| Adulthood (18-64 years) | 81(30.2) | 187 (69.8) |
| Ethnic-minority non-migrant | 50 (22.2) | 175 (77.8) |
| *Missing^2^* | *23 (100.0)* | *0 (0.0)* |
| ***Age group***^3^ | *Χ****^2^=12.2 (4)*** | ***p=0.02*** |
| 18-24 years | 160 (26.6) | 441 (73.4) |
| 25-34 years | 200 (30.2) | 462 (69.8) |
| 35-44 years | 128 (33.2) | 257 (66.8) |
| 45-54 years | 82 (26.6) | 226 (73.4) |
| 55-64 years | 36 (20.6) | 139 (79.4) |
| *Missing^2^* | *1 (100.0)* | *0 (0.0)* |
| ***Sex*** | *Χ****^2^=0.9 (1)*** | ***p=0.36*** |
| Male | 337 (29.3) | 813 (70.7) |
| Female | 270 (27.5) | 712 (72.5) |
| *Missing^2^* | *0 (0.0)* | *0 (0.0)* |
| ***Place of birth***^3^ | *Χ****^2^=175.6 (10)*** | ***p<0.001*** |
| France | 41 (20.8) | 156 (79.2) |
| Italy | 182 (43.7) | 234 (56.3) |
| Spain | 117 (32.9) | 239 (67.1) |
| The Netherlands | 40 (12.1) | 291 (87.9) |
| UK | 58 (14.8) | 333 (85.2) |
| Other Europe | 36 (57.1) | 27 (42.9) |
| Asia & Australasia | 15 (33.3) | 30 (66.7) |
| Sub-Saharan Africa | 42 (37.2) | 71 (62.8) |
| North Africa & Middle East | 19 (38.0) | 31 (62.0) |
| Americas | 50 (37.6) | 83 (62.4) |
| Other | 7 (18.9) | 30 (81.1) |
| *Missing^2^* | *0 (0.0)* | *0 (0.0)* |
| ***Ethnoracial identity*** | *Χ****^2^=5.7 (5)*** | ***p=0.33*** |
| White | 438 (27.4) | 1,162 (72.6) |
| Black | 82 (32.5) | 170 (67.5) |
| Mixed | 21 (25.6) | 61 (74.4) |
| Asian | 20 (31.7) | 43 (68.3) |
| North African | 26 (34.2) | 50 (65.8) |
| Other | 20 (33.9) | 39 (66.1) |
| *Missing^2^* | *0 (0.0)* | *0 (0.0)* |
| ***Fluency (binarized)***^3^ | *Χ****^2^=7.2 (1)*** | ***p=0.007*** |
| Yes | *434 (24.2)* | *1,356 (75.8)* |
| No | *80 (32.1)* | *169 (67.9* |
| *Missing^2^* | *93 (4.4)* | *0 (0.0)* |
| ***Parental social class*** | *Χ****^2^=21.4 (3)*** | ***p<0.001*** |
| Professional | 107 (16.6) | 539 (83.4) |
| Intermediate | 102 (18.4) | 453 (81.6) |
| Working Class | 183 (26.0) | 521 (74.6) |
| Long-term unemployed | 5 (29.4) | 12 (70.6) |
| *Missing^2^* | *210 (9.8)* | *0 (0.0)* |
| ***Living arrangement prior to migration/five years before migration*** | *Χ****^2^=1.6 (2)*** | ***p=0.45*** |
| Alone | 50 (25.0) | 150 (75.0) |
| Family | 267 (21.1) | 997 (78.9) |
| Other | 108 (22.2) | 378 (77.8) |
| *Missing^2^* | *182 (8.5)* | *0 (0.0%)* |
| ***Parental history of psychosis*** | *Χ****^2^=4.7 (1)*** | ***p=0.03*** |
| No | 264 (15.3) | 1,466 (84.7) |
| Yes | 19 (24.6) | 59 (79.6) |
| *Missing^2^* | *324 (15.2)* | *0 (0.0%)* |
| ***Parental history of other mental illness*** | *Χ****^2^=0.9 (1)*** | ***p=0.34*** |
| No | 219 (16.3) | 1,128 (83.7) |
| Yes | 88 (18.1) | 397 (81.9) |
| *Missing^2^* | *300 (14.1)* | *0 (0.0%)* |
| ***Childhood trauma experiences*** | *Χ****^2^=1.6 (3)*** | ***p=0.76*** |
| Quartile 1 (25-28) | 108 (22.8) | 365 (77.2) |
| Quartile 2 (29-33) | 122 (24.4) | 378 (75.6) |
| Quartile 3 (34-42) | 136 (25.8) | 392 (74.2) |
| Quartile 4 (43-102) | 125 (24.4) | 390 (75.7) |
| *Missing^2^* | *116 (5.4)* | *0 (0.0%)* |

Χ^2^: Pearson’s Chi^2^ test

^1^Participants who were missing on at least one exposure or covariate

^2^Item-level missingness, not included in Χ^2^-test comparisons. Percentage is percentage of all participants (N=2,132) missing data on that item

^3^Presented for descriptive purposes and included as auxiliary variables during multiple imputation by chained equations, but not included as part of the covariate adjustment set.
