# Supplementary material for: Age-at-migration, ethnicity and psychosis risk: Findings from the EU-GEI case-control study
Source: PLOS Ment Health. 2024 Oct 2;1(5):e0000134. doi: 10.1371/journal.pmen.0000134 (PMC12798472; doi:10.1371/journal.pmen.0000134)
Supplement: S3 Table — 1Adjusted for gender, parental social class, living arrangement before migration (for migrants) and five years prior to assessment (non-migrants), parental psychosis experience and parental other mental health experience. 2Model 1 + adjustment for ethnoracial identity. *p<0.05, bold. #p = 0.057. ^p = 0.062. (DOCX) [file pmen.0000134.s004.docx]

**S3 Table: Reparameterization of selected logistic regression models with migration during adolescence set as the reference group**

| ***Effect sizes by age-at-migration*** | **Whole sample**  **Model 1**  **aOR (95% CI)^1^** | **Whole sample**  **Model 2**  **aOR (95% CI)^2^** | **Black**  **Model 1**  **aOR (95% CI) ^1^** | **North African**  **Model 1**  **aOR (95% CI) ^1^** |
| --- | --- | --- | --- | --- |
| White majority non-migrant | **0.31 (0.18-0.53)*** | **0.52 (0.30-0.90)*** | **0.15 (0.07-0.33)*** | **0.06 (0.01-0.53)*** |
| Infancy (0 to 4 years) | 0.62 (0.36-1.08) | 0.70 (0.41-1.17) | 0.49 (0.16-1.50) | - |
| Childhood (5 to 10 years) | 0.64 (0.26-1.57) | 0.68 (0.27-1.70) | **0.30 (0.12-0.74)*** | 0.23 (0.04-1.36) |
| Adolescence (11 to 17 years) | 1 | 1 | 1 | 1 |
| Adulthood (18 to 64 years) | **0.52 (0.28-0.98)*** | 0.61 (0.33-1.12) | 0.41 (0.17-1.03)^#^ | 0.20 (0.04-1.09)^ |
| Ethnic minority non-migrant | 0.69 (0.39-1.21) | 0.59 (0.32-1.07) | **0.33 (0.11-0.91)*** | **0.22 (0.05-0.91)*** |

^1^Adjusted for gender, parental social class, living arrangement before migration (for migrants) and five years prior to assessment (non-migrants), parental psychosis experience and parental other mental health experience

^2^Model 1 + adjustment for ethnoracial identity

*p<0.05, **bold**

^#^p=0.057

^^^p=0.062
