# Supplementary material for: Age-at-migration, ethnicity and psychosis risk: Findings from the EU-GEI case-control study
Source: PLOS Ment Health. 2024 Oct 2;1(5):e0000134. doi: 10.1371/journal.pmen.0000134 (PMC12798472; doi:10.1371/journal.pmen.0000134)
Supplement: S4 Table — 1Adjusted for gender, parental social class, living arrangement before migration (for migrants) and five years prior to assessment (non-migrants), parental psychosis experience and parental other mental health experience. 2Model 1 + adjustment for ethnoracial identity. *p<0.05, bold. #p = 0.053. ^p = 0.057. (DOCX) [file pmen.0000134.s005.docx]

**S4 Table: Multivariable regression results from complete case analyses (N=1,525)**

| ***Effect sizes by age-at-migration*** | **Whole sample**  **Unadjusted**  **OR (95% CI)** | **Whole sample**  **Model 1**  **aOR (95% CI)^1^** | **Whole sample**  **Model 2**  **aOR (95% CI)^2^** |  |  |  |
| --- | --- | --- | --- | --- | --- | --- |
| White majority non-migrant | 1 | 1 | 1 |  |  |  |
| Infancy (0-4 years) | **2.99 (2.02-4.42)*** | **2.79 (1.32-5.92)*** | 1.62 (0.86-3.07) |  |  |  |
| Childhood (5-10 years) | **2.23 (1.11-4.48)*** | 1.86 (0.83-4.13) | 1.13 (0.43-3.07) |  |  |  |
| Adolescence (11-17 years) | **3.15 (1.63-6.08)*** | **3.10 (1.75-5.47)*** | 1.74 (0.89-3.40) |  |  |  |
| Adulthood (18-64 years) | 1.39 (0.89-2.18) | 1.58 (0.99-2.52)^#^ | 1.08 (0.56-2.13) |  |  |  |
| Ethnic minority non-migrant | **2.63 (2.27-3.04)*** | **2.07 (1.81-2.37)*** | 1.03 (0.55-1.91) |  |  |  |
|  |  |  |  |  |  |  |
| *Stratified results by ethnoracial identity* | **White**  **aOR (95% CI)^1^** | **Black**  **aOR (95% CI) ^1^** | **Mixed**  **aOR (95% CI) ^1^** | **Asian**  **aOR (95% CI) ^1^** | **North African**  **aOR (95% CI) ^1^** | **Other**  **aOR (95% CI) ^1^** |
| White majority non-migrant | 1 | 1 | 1 | 1 | 1 | 1 |
| Infancy (0-4 years) | 2.00 (0.61-6.50) | **4.53 (2.01-10.24)*** | 1.30 (0.06-30.15) | - | - | 0.88 (0.10-7.61) |
| Childhood (5-10 years) | 1.60 (0.54-4.74) | 2.04 (0.60-6.93) | 3.75 (0.18-78.88) | - | 3.53 (0.22-55.52) | - |
| Adolescence (11-17 years) | 2.21 (0.33-14.70) | **6.13 (2.36-15.90)*** | - | 0.96 (0.20-4.48) | 10.42 (0.92-117.70)^ | 3.29 (0.20-54.90) |
| Adulthood (18-64 years) | 0.96 (0.45-2.07) | **2.39 (1.66-3.43)*** | 0.60 (0.13-2.68) | 2.58 (0.59-11.17) | 3.42 (0.89-13.20) | 2.06 (0.63-6.81) |
| Ethnic minority non-migrant | - | **2.02 (1.22-3.36)*** | **3.48 (1.67-7.27)*** | 1.30 (0.55-3.04) | 2.39 (0.44-13.09) | 1.47 (0.33-6.59) |

^1^Adjusted for gender, parental social class, living arrangement before migration (for migrants) and five years prior to assessment (non-migrants), parental psychosis experience and parental other mental health experience

^2^Model 1 + adjustment for ethnoracial identity

*p<0.05, **bold**

^#^p=0.053

^^^p=0.057
