# Supplementary material for: Age-at-migration, ethnicity and psychosis risk: Findings from the EU-GEI case-control study
Source: PLOS Ment Health. 2024 Oct 2;1(5):e0000134. doi: 10.1371/journal.pmen.0000134 (PMC12798472; doi:10.1371/journal.pmen.0000134)
Supplement: S1 Text — (DOCX) [file pmen.0000134.s006.docx]

# *Lived Experience Advisory Group input*

# The lived experience advisory group was recruited at the beginning of the study to inform the first author’s wider PhD project. This group was made up of four people (one male, three females) who had direct migration history and lived experience of psychosis and/or mental health problems. All of the group members were from ethnic minority backgrounds. The group met at regular times to discuss project progress and the meetings consisted of an update presentation by the first author and potential discussion points and time to feedback. The group also received monthly updates of the project via email. Through these meeting formats, the group were involved in the analysis plan and design of the direct acyclic graph (DAG), suggesting potential confounders and mediators in the association between age at migration and psychosis risk on the DAG. During the data cleaning and coding process, the group discussed factors that would limit the analysis (for example controlling for pre-migratory social class in the context of migration in childhood and adolescence was deemed to not be an appropriate variable and the group felt it was more relevant to use parental social class as a confounder). Such suggestions were checked for consistency with epidemiological theory on causal inference, and retained where this condition was met. The first author presented and discussed missing data patterns with the group, and plans to address this via multiple imputation. The first author presented the complete case results and potential explanations to explain statistically non-significant results and sample sizes. After imputing the data, the group met again and discussed the associations and stratified results. The first author shared potential discussion points and previous literature that could explain the increased risk of psychosis associated with adolescent migration. The group were able to feedback on these ideas as well as use their experiential expertise to explain why some ethnoracial ~~ic~~ minority groups might be at greater risk of psychosis when migrating in adolescence. Those in the group who had migrated during adolescence discussed their experiences of migration and onset of psychosis and social determinants that influenced their mental health and used this to inform how the results from this study might be contextualised to adolescent migration and psychosis onset. Though the group were not directly involved in coding or analysis, their experiential expertise informed the process throughout and the first author used their experiences to review literature and discuss the results of the study.
