# Supplementary material for: Age-at-migration, ethnicity and psychosis risk: Findings from the EU-GEI case-control study
Source: PLOS Ment Health. 2024 Oct 2;1(5):e0000134. doi: 10.1371/journal.pmen.0000134 (PMC12798472; doi:10.1371/journal.pmen.0000134)
Supplement: S2 Text — (DOCX) [file pmen.0000134.s007.docx]

# *Missing data*

We used Multiple Imputation by Chained Equations (MICE) to handle missing data. In addition to all models included in the main regression model, we include four auxiliary variables to aid imputation – EU-GEI site, birthplace, age group at first contact and language fluency. The EU-GEI site variable was a categorical variable that included the 16 European sites of the EU-GEI study (UK: South London, Cambridgeshire; The Netherlands: Amsterdam, Gooda & Voorhout; Spain: Madrid, Barcelona, Valencia, Oviedo, Santiago, Cuenca; France: Val-de-Marne (Creteil), Puy-de-Dôme, Maison Blanche (Paris); Italy: Veneto, Bologna, Palermo). Region of ~~B~~birth ~~place~~ was defined by country (for EU-GEI original sites i.e. Brazil France, Italy, Spain, The Netherlands, UK) or otherwise broad region of birth including: other Europe, Australasia (including the Pacific Islands), Asia, Sub-Saharan Africa, North Africa or the Middle East, the Americas, or Other). Age-group-at-first-contact was based on age of first presentation (in cases) or age at study consent (in controls) and classified as 16-24, 25-34, 35-44, 45-54, 55-64 years old). Self-reported language fluency in the majority language was rated on a score of 1 (not fluent at all) to 10 (very fluent).

It was necessary to use passive imputation methods following our MICE routine for two sets of exposure or covariate variables: categorical age-at-migration, and interaction terms for categorical age-at-migration by ethnoracial identity~~icity~~. The reasons for this and methodology are described below.

Categorical age-at-migration: Logically, first generation migrants who were missing migration age would be classed as ‘soft missing’ (i.e. having a missing value for which an imputed value is valid and desired), whereas all non-migrants (White majority non-migrants, or second generation migrants) who have never migrated in this context would be considered ‘hard missing’ (i.e. not applicable to have a migration age value). However, direct imputation of our categorical age-at-migration variable (which included White majority non-migrants and second generation migrants as separate categories, in addition to levels of age-at-migration for first generation migrants) would have led to some first generation migrants with a missing migration age value potentially being assigned to receiving an invalid imputed value, as either the White group or second generation categories, rather than to a valid age-at-migration category (infancy, childhood, adolescence, adulthood). Twenty-three first generation migrants were (soft) missing migration age.

To overcome this issue, we created two age-at-migration variables prior to imputation (“AOM1”, “AOM2”). AOM1 was the original categorical age-at-migration variable (levels: White Majority, Second Generation, or migration during: infancy, childhood, adolescence, adulthood), containing known values for all groups except for 23 first generation migrants soft missing their migration age. AOM2 was created for the whole sample, but restricted to the age-at-migration categories to which first generation migrants could logically belong (infancy, childhood, adolescence, adulthood). All participants with missing values on AOM2 (i.e. N=23 first generation migrants with a soft missing value; N=2,043 non-migrants hard missing on this variable) were set as soft missing on AOM2. We then imputed soft missing values for AOM1 (N=23) and AOM2 (N=2,066) during the MICE routine across 50 imputed datasets. After imputation, we passively created our categorical age-at-migration variable for use as the exposure variable in our analyses. Here, we created a new variable (“AOM3”) based on the final values of AOM1 and AOM2. AOM1 provided the correct values (as observed) for all but the 23 first generation migrants initially soft missing migration age. For these 23 participants, we replaced their migration age category in AOM3 with their imputed values from AOM2.

Categorical age-at-migration by ethnoracial identity~~icity~~: To examine whether the association between age-at-migration and psychosis differed by ethnoracial identity ~~icity~~ would routinely require either (a) stratification by ethnoracial ~~ic~~ group and/or (b) fitting and testing of an interaction term between age-at-migration and ethnoracial~~ic~~ group. However, in our dataset both our age-at-migration and ethnoracial ~~ic~~ group variables had a ~~the highly~~ collinear reference category~~ies~~ (the White group for ethnoracial identity variable ~~icity~~ or the White majority non-migrant group for age-at-migration) leading to difficulties in estimation of the model. To overcome this, we performed stratified comparisons of the association between age-at-migration and psychosis for each ethnoracial ~~ic minority~~ group compared with the White Majority group. Using the imputed data derived on age-at-migration above (i.e. “AOM3”), we passively created a reference group for these stratified analyses (the White group with no migration history). Then, in a series of stratified regression analyses for each ethnoracial ~~ic~~ group (White, Black, North African, Asian, Mixed, Other), we compared the odds of psychosis associated with ~~each~~ age-at-migration to the odds of psychosis in this reference group ~~(i.e. odds ratios for migration in infancy, childhood, adolescence and adulthood for the Black group compared with the White Majority, Non-Migrant group)~~. Some estimates for the North African group following multiple imputation may have been biased due to variation in estimation samples between different imputed datasets, likely driven by a sparsity of data.

The MICE model imputed all variables according to their type, using logistic regression models as appropriate for binary, ordinal or nominal data. To aid modelling, some of these variables were treated as continuous predictors of other missing variables (for example, fluency, AOM2, trauma, parental SES, age group were entered as continuous predictors). The White ethnoracial ~~ic~~ group, Sao Paolo and Brazil were treated as the reference categories for ethnoracial identity~~icity~~, EU-GEI setting and country of birth, respectively, as these were the most common responses in our dataset.

Appropriate regression models (logistic regression, ordinal logistic regression, multinomial logistic regression) were used during multiple imputation according to the type of variable. MICE was fitted using an augmented regression approach to add extra observations with very low weights to prevent perfect prediction and aid model fitting. Adding low weights to these observations has a negligible effect on the results, but prevents perfect prediction. Fifty datasets were imputed and analyses were conducted using these datasets using Rubin’s Rule
